# IL‐7 is expressed in malignant mesothelioma and has a prognostic value

**DOI:** 10.1002/1878-0261.13310

**Published:** 2022-09-10

**Authors:** Hoa‐Le Mai, Sophie Deshayes, Thi‐Van‐Ha Nguyen, Virginie Dehame, Anne‐Laure Chéné, Sophie Brouard, Christophe Blanquart

**Affiliations:** ^1^ CHU Nantes, INSERM, Center for Research in Transplantation and Translational Immunology, UMR 1064 Nantes Université Nantes France; ^2^ Immunology Graft Oncology Labex IGO Nantes France; ^3^ Nantes Université, Inserm UMR 1307, CNRS UMR 6075, Université d'Angers CRCI2NA Nantes France; ^4^ Service de pneumologie, L'institut du thorax, Hôpital Guillaume et René Laennec CHU Nantes Nantes France

**Keywords:** biomarker, IL‐7, IL‐7R, mesothelioma, pleural fluids

## Abstract

Malignant pleural mesothelioma (MPM) is an aggressive cancer mainly related to asbestos exposure. Despite recent therapeutic advances, notably immunotherapies, the benefit remains limited and restricted to a small percentage of patients. Thus, a better understanding of the disease is needed to identify new therapeutic strategies. Recently, interleukin 7 receptor (IL‐7R) has been described as being expressed by MPM cells and associated with poorer patient survival. Thus, the aim of this work was to study the IL‐7R/IL‐7 pathway in MPM using patient samples. We found that, although more than 40% of MPM cells expressed IL‐7R, IL‐7 had no effect on their intracellular signaling. Accordingly, the addition of IL‐7 to the culture medium did not affect MPM cell growth. Using The Cancer Genome Atlas (TCGA) database, we showed that high *IL7* gene expression in MPM tumors was associated with a higher overall patient survival and an induction of genes involved in the immune response. In pleural effusions (PEs), we found that IL‐7 concentration was not a good diagnostic biomarker. However, we observed that high IL‐7 levels in PEs were associated with shorter survival of MPM patients, but not of lung cancer patients. The prognostic value of IL‐7 was also conserved when only patients with epithelioid mesothelioma, the most common histological type of MPM, were analyzed. Taken together, our study suggests that, although the IL‐7R/IL‐7 signaling pathway is not functional in MPM cells, IL‐7 expression in PEs may have prognostic value in MPM patients.

## Graphical abstract

Tumor expression of interleukin‐7 receptor (IL‐7R) has been reported to be associated with poor prognosis in malignant pleural mesothelioma (MPM), a highly aggressive cancer. We herein confirm that MPM cells can express IL‐7R and secrete IL‐7 but they do not respond to IL‐7/IL‐7R signaling. Importantly, we show that MPM patients with high IL‐7 level in pleural effusion have poorer survival.

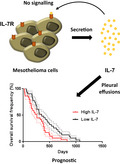

**Abbreviations**
ADCA: lung adenocarcinoma
APC: allophycocyanin
AUC: area under the curve
BM: biphasic mesothelioma
BPE: benign pleural effusion
CD127: IL‐7Rα
CD132: IL‐2Rγ
DM: diffuse mesothelioma
EM: epithelioid mesothelioma
FCS: fetal calf serum
GSEA: gene set enrichment analysis
IL‐7R: interleukin 7 receptor
JAK: Janus kinase
MC: primary mesothelial cells
MFI: mean fluorescence intensity
M‐MLV: Moloney murine leukemia virus
MPM: malignant pleural mesothelioma
NK cell: natural killer cell
NSCLC: nonsmall cell lung cancer
PBMC: peripheral blood mononuclear cell
PE: phycoerythrin
PEs: pleural effusions
RNA‐Seq: RNA sequencing
ROC: receiver operating characteristic
RT‐PCR: real‐time polymerase chain reaction
SEM: standard error of the mean
SM: sarcomatoid mesothelioma
SMRP: soluble mesothelin‐related peptide
STAT: signal transducer and activator of transcription
TCGA: The Cancer Genome Atlas
TSLP: thymic stromal lymphopoietin
VEGF‐D: vascular endothelial growth factor D

## 1 Introduction

Malignant pleural mesothelioma (MPM) is a very aggressive cancer that develops from the mesothelial cells of the pleura, usually associated with previous asbestos exposure. Three main histological subtypes of MPM have been described, the epithelioid (50–60%), sarcomatoid (10–20%), and biphasic subtype [1]. Because of unspecific clinical and radiological signs, mainly related to pleural effusion, MPM is often diagnosed at an advanced stage of the disease, 30–40 years after asbestos exposure. The prognosis of MPM is among the poorest of all cancers, with around 12 months median survival and < 10% 5‐year survival rate. The first line regimen, cisplatin‐pemetrexed combination only improves median survival by 3 months; the combination of bevacizumab with first line treatment only further improves survival for three additional months [2]. Despite encouraging results, immunotherapy approaches only improve outcome of the disease in some patients [3]. It is now necessary to understand the determinants of sensitivity and resistance to therapies as well as to look for potential prognostic biomarkers to anticipate and improve the clinical care of this pathology.

Recently, Ujiie *et al*. performed immunohistochemical staining of more than 200 pleural tumor specimens from patients diagnosed with malignant pleural mesothelioma and found that high tumor expression of IL‐7R was associated with shorter overall survival [4], suggesting a potential role of the IL‐7 signaling pathway in the progression of this disease. Interleukin‐7 (IL‐7) is an important cytokine that controls lymphopoiesis [5]. The receptor of IL‐7 (IL‐7R) is formed by 2 chains, a common gamma chain (IL‐2Rγ, also called CD132) which is a component of the receptors of several cytokines: IL‐2, 4, 7, 9, 15, and 21 [6] and an alpha chain (IL‐7Rα, also called CD127) which is a component of the receptor of either IL‐7 or thymic stromal lymphopoietin (TSLP). IL‐7 or IL‐7R knockout mice lack T and B cells but have normal NK cells [7, 8] and the mutation of IL‐7Rα in humans leads to reduced T cell number, whereas B cell number is not affected [9]. In humans, several lines of evidence suggest the role of the IL‐7 signaling pathway in autoimmune, chronic, and inflammatory diseases [10, 11]. The IL‐7 signaling pathway is also involved in many types of cancers, including hematologic malignancies as well as solid tumors. Gain‐of‐function mutations in the IL‐7Rα is present in about 10% of childhood T‐cell acute lymphoblastic leukemia, causing constitutive activation of the receptor and its downstream signaling leading to leukemic transformation [12]. The IL‐7Rα transcript and protein are also expressed in several solid tumor cell lines such as lung, breast, and brain cancer [13]. IL‐7/IL‐7R signaling was shown to induce lung and breast tumor growth and lymphangiogenesis via upregulation of vascular endothelial growth factor D (VEGF‐D) [14, 15]. High expression of IL‐7R in lung adenocarcinoma was shown to be associated with poor survival [16].

The objective of this study was to evaluate the IL‐7/IL‐7R pathway in mesothelioma using a collection of samples from patients. We characterized the expression and the functionality of IL‐7R in a collection of MPM cell lines established from pleural effusions of patients, we measured IL‐7 in a collection of pleural effusions and serum from patients and we analyzed Tumor Cancer Genome Atlas (TCGA) dataset to confirm our results.

## 2 Materials and methods

### 2.1 Drugs and cytokine

Tofacitinib (JAK3 inhibitor) and ruxolitinib (JAK1/2 inhibitor) were purchased from Selleckchem and IL‐7 from R&D System (Minneapolis, MN, USA).

### 2.2 Collection of mesothelioma cell lines and pleural effusions

The mesothelioma (MPM) (*n* = 22) (GSE134349), including Meso11, Meso13, Meso34, Meso35, Meso36, Meso45, Meso52, Meso62, Meso152, Meso163, and Meso182, and lung adenocarcinoma (ADCA) (*n* = 7) cell lines were established in our laboratory from pleural fluids (PEs) of patients (Table S1) [17]. All cell lines were maintained in RPMI‐1640 medium (Gibco, Thermo Fisher Scientific, Waltham, MA, USA) supplemented with 2 mm L‐glutamine, 100 IU·mL^−1^ penicillin, 0.1 mg·mL^−1^ streptomycin, and 10% heat‐inactivated fetal calf serum (FCS) (Gibco) and cultured at 37 °C in a 5% CO_2_ atmosphere. The primary mesothelial cells (MC) were isolated from pleural surgery samples [18].

PEs from patients with a suspected mesothelioma were aseptically collected by thoracocentesis at the Laënnec Hospital (St‐Herblain, France) between 1998 and 2016. Our collection included 80 PE from MPM patients, 108 PE from other neoplasia patients, and 24 benign pleural effusions (BPE). Samples were centrifuged at 1000 ***g*** in a Heraeus Multifuge for 20 min at +4 °C and supernatants were aliquoted and stored at −80 °C. Serum samples were also collected at the Laennec Hospital, aliquoted and stored at −80 °C. These methodologies were previously described by Gueugnon *et al*. [17]. Diagnoses were established by both fluid cytology and immunohistochemical staining of pleural biopsies performed by the pathology department at Laennec Hospital (St‐Herblain, France) and then externally confirmed by Mesopath, the French panel of pathology experts for the diagnosis of mesothelioma. Patients with mesothelioma were then treated with platinum‐based chemotherapy when the patients' general condition was correct. For those with advanced disease, palliative medical care was provided. Samples were collected in accordance with the standards established by the Declaration of Helsinki. All recruited patients had received no prior anticancer therapy and gave signed informed consent. All the collected samples and the associated clinical information were registered in a database (DC‐2014‐2206) validated by the French ministry of research. Study was approved by local ethical committee (CPP Ouest‐IV‐Nantes).

### 2.3 RNA isolation and real‐time PCR from cell lines

Total RNA was isolated from cultured mesothelioma cell lines using RNeasy Mini Kit (Qiagen) according the manufacturer's instruction. One microgram of total RNA was reversed‐transcribed into cDNA using Moloney murine leukemia virus (M‐MLV) reverse transcriptase (Invitrogen, Thermo Fisher Scientific, Waltham, MA, USA). Quantitative real‐time PCR (RT‐PCR) was then performed using Applied Biosystem TaqMan® Gene Expression assays (Thermo Fisher Scientific, Waltham, MA, USA). The following TaqMan® probes were used: IL‐7 (Hs00174202_m1), IL‐7Rα (Hs00233682_m1), TSLP (Hs00263639_m1), TSPLR (Hs00845692_m1), IL‐2RG (Hs00415671_m1), JAK1 (Hs01026983_m1), JAK3 (Hs00354555_m1), STAT1 (Hs01013996_m1), STAT3 (Hs00374280_m1), STAT5A (Hs00559637_g1), STAT5B (Hs00560026_m1), and HPRT (Hs99999909_m1). RT‐PCR was performed in duplicate in 96‐well RT‐PCR plate with 10 μL final reaction mix per well containing 2 μL cDNA, 2.5 μL H2O, 0.5 μL TaqMan probe (20×), and 5 μL TaqMan Fast Advanced Master Mix (2×). The RT‐PCR was run for 40 cycles using the ViiA 7 Real‐Time PCR system (Thermo Fisher Scientific). Relative gene expression was calculated by the Δ*C*_t_ method with HPRT as a house‐keeping gene.

### 2.4 Flow cytometry

Mesothelioma cell lines were stained for surface markers with the following fluorescence‐conjugated monoclonal antibodies (mAbs): PE mouse anti‐human CD127 (clone HIL‐7R‐M21), APC rat anti‐human CD132 (clone TUGh4), PE mouse anti‐human TSLPR (clone 1F11/TSLPR), PE mouse IgG1 (kappa) isotype control (clone MOPC‐31C), and APC rat IgG2b (kappa) isotype control (clone A95‐1). All mAbs were from BD Biosciences (BD Bioscience France, Le Pont‐de‐Claix, France) except anti‐CD132 was from BioLegend (San Diego, CA, USA). Data were acquired using a BD LSR II flow cytometer and then analyzed with flowjo software (Flowjo, Ashland, OR, USA). The relative mean fluorescence intensity (MFI) of CD127 and CD132 was defined as the ratio of the MFI of CD127 and CD132 to the MFI of the respective isotype control. STAT5 phosphorylation was detected by flow cytometry following the Phosflow staining protocol provided by BD Biosciences. Briefly, MPM cell lines or human Peripheral blood mononuclear cells (PBMCs; positive control) were incubated with human IL‐7 for 15 min at 37 °C, fixed with BD Cytofix for 30 min on ice, stained with BV421 anti‐CD3 mAb (for PBMCs only), incubated with BD Perm buffer III, and then stained with Alexa Fluor 647 anti‐pSTAT5 (clone 47/Stat5 pY694). Cells were washed twice with BD Perm/Wash buffer after each step.

### 2.5 Viability assay

MPM cells were seeded at 5 × 10^3^ cells per well of 96‐well plate in 180 μL of culture medium. After 24 h, 20 μL of 10 times concentrated compounds were added for 72 h. Viability was determined using Cell Titer‐Glo kit (Promega, Madison, WI, USA) according to manufacturer's recommendations.

### 2.6 Western‐Blot

MPM cell lines were seeded in six‐well plates at a density of 0.5 × 10^6^ cells per well. Fresh human PBMC, prepared by Ficoll from healthy blood donor, were seeded at 10^6^ cells per well of six‐well plates. Thereafter, cells were treated or not with 10 ng·mL^−1^ of IL‐7 (R&D System) for 15 min. Cells were lysed in RIPA buffer containing a Protease Inhibitor Cocktail (Sigma‐Aldrich, Saint‐Louis, MO, USA) and denatured at 95 °C for 5 min in Laemmli buffer with 10% β‐mercaptoethanol. Then, 10 μg of proteins for cellular lysate were separated by sodium dodecyl sulfate polyacrylamide gel electrophoresis on 8% gels and transferred to polyvinylidene difluoride membranes. Blots were incubated with anti‐STAT5 (Clone 89, BD Biosciences) or anti‐p(Y694)‐STAT5 (Clone 47, BD Biosciences) at 0.5 μg·mL^−1^ in PBS‐T 0.1% + BSA 5% followed by incubation with HRP‐coupled secondary antibodies (Jackson ImmunoResearch, West Grove, PA, USA). Proteins were revealed using Enhanced Chemiluminescence Detection ECL (Bio‐Rad, Hercules, CA, USA).

### 2.7 Analysis of the cancer genome atlas dataset

All RNAseqv2 samples from The Cancer Genome Atlas (TCGA)‐MESO dataset (*n* = 87 patients) are available on the Broad's Genome Data Analysis Centre (http://gdac.Broad‐institute.Org/). Gene expressions as RNA‐seq by expectation maximization values (RSEM values) were analyzed. Clinical data for these samples were downloaded from FireBrowse (http://firebrowse.Org; version 2018_02_26 for MESO). The pathway enrichment analysis was performed using Gene Set Enrichment Analysis (GSEA) and KEGG gene sets.

### 2.8 ELISA assay

IL‐7 titrations were performed with the Human IL‐7 Quantikine ELISA kit (R&D Systems) and SMRP was measured using Mesomark® kit (Fijirebio France, Les Ulis, France) following the manufacturers' recommendations. For IL‐7 measurements, samples were not diluted. For SMRP measurements, samples were diluted at 1 : 1000.

### 2.9 Data and statistical analyses

Comparisons were performed using nonparametric Mann–Whitney *t*‐test. Log‐rank Mantel–Cox test was used for survival analyses. Correlations were evaluated using nonparametric Spearman test. All statistical analyses were performed using graphpad prism (prism V.6 for Windows) (GraphPad Software, San Diego, CA, USA).

## 3 Results

### 3.1 IL‐7R and IL‐7 are expressed by MPM cell lines

**Fig. 1:**
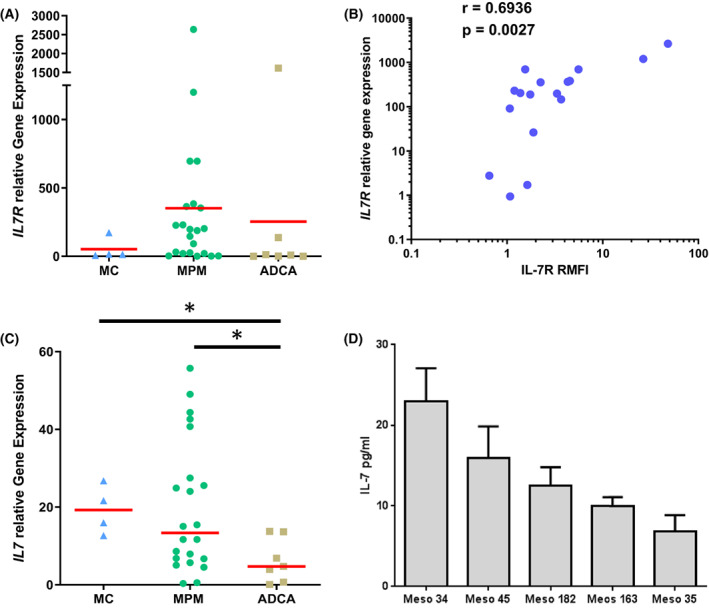
Analysis of IL‐7R and IL‐7 expression in MPM cells. (A, C) mRNA expression of *IL7R* (A) and *IL7* (C) measured by RT‐PCR in MPM (*n* = 22), ADCA (*n* = 7), and primary mesothelial cells (*n* = 4). (B) Correlation between *IL7R* mRNA expression and IL‐7Rα cell surface expression measured by flow cytometry in MPM cells (*n* = 17). Spearman test. (D) IL‐7 secretion by MPM cells as measured by ELISA of cell culture supernatants. Red horizontal bars represent median expression. Bar graphs represent mean ± SEM of three independent experiments. (A, C) Mann–Whitney *t*‐test. *P < 0.05. ADCA, lung adenocarcinoma; MC, mesothelial cell; MPM, malignant pleural mesothelioma.

We first measured *IL7R* and *IL7* gene expressions in a collection of MPM (*n* = 22) and lung ADCA cell lines (*n* = 7), and in primary mesothelial cells (MC) (*n* = 4) using RT‐PCR. *IL7R* expression was heterogeneous in MPM cells compared to MC (Fig. 1A). Two populations were observed in MPM cells, one with no *IL7R* expression and one with high IL7R expression compared to MC. In lung ADCA cell lines, *IL7R* expression was low except for one cell line. *IL7R* mRNA expression was correlated with CD127 protein expression at the surface of MPM cells (Fig. 1B). Overall, 57.10% (12/21) of the MPM cell lines expressed CD127 on the cell surface (Table 1). *IL7* mRNA expression was significantly higher in MPM cells and MC compared to ADCA cells (*P* = 0.035 and *P* = 0.024, respectively) (Fig. 1C). Next, we measured the concentration of IL‐7 in the culture supernatant of some MPM cell lines. We confirmed that these cells can secrete IL‐7 (Fig. 1D) and the levels of IL‐7 secretion correlates with the levels of IL‐7 mRNA expression (Fig. S1). However, we did not find a correlation between the mRNA expression of IL7R and IL‐7 in MPM cell lines (Fig. S2).

**Table 1:** Percent of MPM cell lines expressing CD127 and or CD132.

CD127^+^	CD127^+^ and CD132^+^
57.10% (12/21)	42.85% (9/21)

### 3.2 MPM cell lines also express IL‐2RG but not TLSPR

**Fig. 2:**
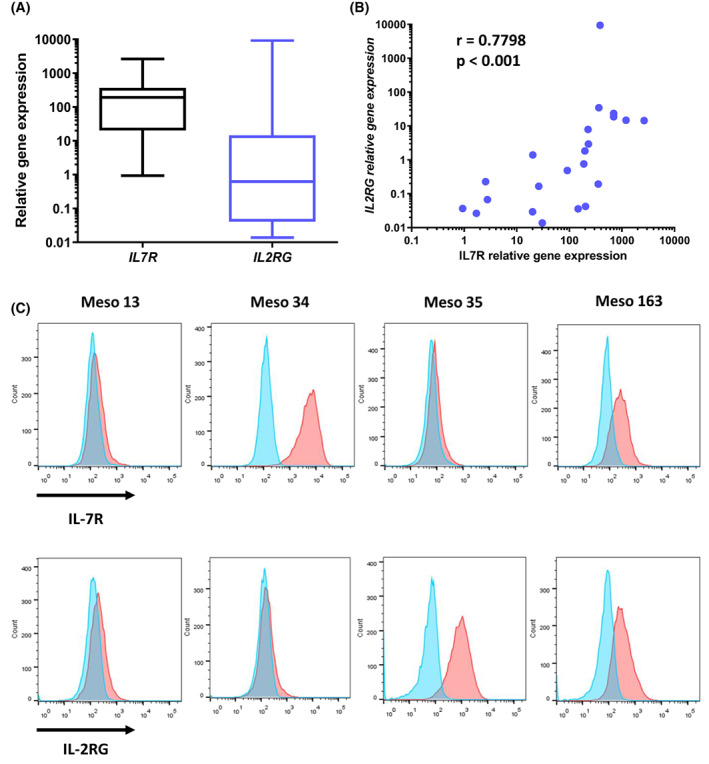
Analysis of IL‐7R and IL2RG expression in MPM cells (*n* = 22). (A) mRNA expression of *IL7R* and *IL2RG* as measured by RT‐PCR in MPM cells. Graphs are whiskers plots (min to max). The line in the middle of the box is plotted at the median. (B) Correlation between *IL7R* and *IL2RG* mRNA expression in MPM cells. Spearman test. (C) MPM cell surface expression of IL‐7R and IL‐2RG as determined by flow cytometry. Blue: Isotype controls, red: Anti‐IL‐7Rα (upper) or anti‐IL‐2Rγ (lower) antibody. MPM, malignant pleural mesothelioma.

Since IL‐7R is composed of two chains, CD127 and CD132 (*IL2RG* gene), we measured the expression of *IL2RG* gene in MPM cell lines using RT‐PCR. We observed that the expression of *IL2RG* was lower than that of *IL7R* in MPM cells (Fig. 2A) and there was a positive correlation between *IL7R* and *IL2RG* mRNA expression (Fig. 2B and Table S2). Next, we studied the expression of CD127 and CD132 protein on the cell membrane by flow cytometry and found that they were not always coexpressed. As illustrated in Fig. 2C, Meso 13 cell line expressed neither CD127 nor CD132, Meso 34 expressed CD127 only, Meso 35 expressed CD132 only, whereas Meso 163 expressed both markers. Overall, 42.85% of MPM cell lines expressed both CD127 and CD132 on the cell surface (Table 1). Because CD127 can also interact with TSLPR to form the receptor of TSLP, we measured the expression of TSLPR in MPM cell lines. We observed a correlation between *IL7R* and *TSLPR* at the mRNA level (spearman *r* = 0.567, *P* = 0.0059) (Table S2). However, only 1/12 cell lines tested expressed TSLPR protein on the cell surface (data not shown).

### 3.3 Absence of IL‐7 signaling in MPM cell lines

**Fig. 3:**
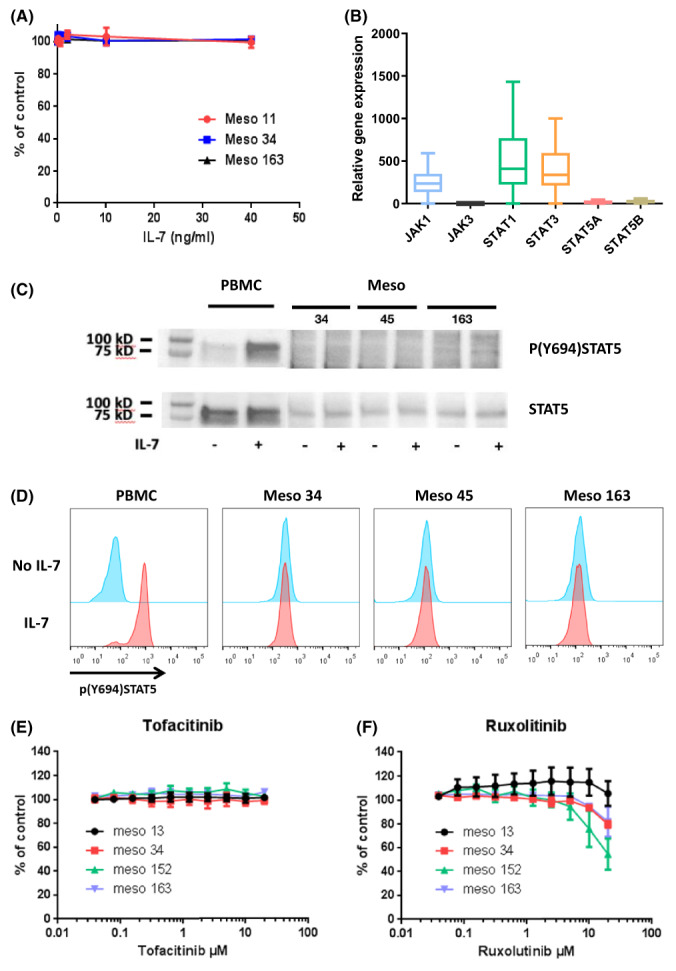
Functional evaluation of the IL‐7R/IL‐7 signaling pathway in MPM. (A) CD127^+^/CD132^+^ cells (Meso163), CD127^−^ cells (Meso11), and CD127^+^/CD132^−^ cells (Meso34) were incubated with increasing doses of IL‐7 for 72 h and cell viability was measured. Results represent means ± SEM of three independent experiments. (B) Expression of genes involved in IL‐7 signaling was measured using RT‐qPCR in MPM cell lines (*n* = 21). Graphs are whiskers plots (min to max). The line in the middle of the box is plotted at the median. (C, D) CD127^+^/CD132^+^ cells (Meso163), CD127+/CD132^−^ cells (Meso45) and CD127^+^/CD132^−^ cells (Meso34) or PBMC were incubated with IL‐7 for 15 min and then STAT5 phosphorylation was evaluated using western blot (C) or flow cytometry (D). Results are representative of two independent experiments. (E, F) MPM cells were treated with increasing doses of tofacitinib (JAK3i) (E) or ruxolitinib (JAK1/2i) (F) for 72 h and cell viability was measured. Results represent means ± SEM of three independent experiments. MPM, malignant pleural mesothelioma; PBMC, peripheral blood mononuclear cells.

Previous studies have shown that IL‐7 can act on cancer cells in an autocrine manner to increase cell proliferation [19]. Therefore, we evaluated the effect of IL‐7 on the growth of different MPM cell lines: CD127^+^/CD132^+^ cells (Meso163), CD127^−^/CD132^−^ cells (Meso11), and CD127^+^/CD132^−^ (Meso34). No effect of IL‐7 was observed irrespective of the expression of CD127 and CD132 (Fig. 3A and Fig. S3A,B). In order to understand the absence of effect of IL‐7 on MPM cell growth, we measured the expression of genes involved in IL‐7 signaling using RT‐PCR. We found that the expression of the major genes involved in the JAK/STAT signaling pathway including JAK1, JAK3, STAT1, STAT3, STAT5a, and STAT5b was very low in MPM cell lines (Fig. 3B and Fig. S3C,D) compared to PBMCs (Fig. S4), the main type of cells highly responsive to IL‐7 stimulation. Next, we performed western blot to investigate whether IL‐7 can induce STAT5 phosphorylation in MPM cell lines. We confirmed that MPM cell lines only weakly expressed STAT5 (Fig. S5A) and the addition of IL‐7 did not induce STAT5 phosphorylation in any tested cell lines, including Meso 163 which strongly expressed both CD127 and CD132. As a positive control, PBMCs strongly expressed STAT5 that was phosphorylated in the presence of IL‐7 (Fig. 3C and Fig. S5A). To confirm these results, we stimulated 8 MPM cell lines with IL‐7 and measured STAT5 phosphorylation using flow cytometry (Fig. 3D and Fig. S6). As observed in western‐blot, IL‐7 induced STAT5 phosphorylation in PBMCs but not in MPM cell lines. In order to exclude a constitutive activation of JAK pathways, which could contribute to the absence of IL‐7 effect on cell growth, we cultured 4 MPM cell lines (Meso 13, 34, 152, and 163) with increasing concentrations of either a JAK3 inhibitor (tofacitinib) or a JAK1/2 inhibitor (ruxolitinib). Those cell lines express JAK1 at both the mRNA (Fig. S3C) and protein (Fig. S5B) levels. On the contrary, they barely express JAK3 mRNA (Fig. S3D) and JAK3 protein was not detectable by western blot (data not shown). Concordantly, tofacitinib had no effect on cell growth (Fig. 3E), whereas ruxolitinib reduced cell growth in three of the four tested cell lines (19%, 46%, and 19% for Meso 34, 152, and 163, respectively) (Fig. 3F and Table S3). Thus, JAK1 seemed to be activated and to contribute to the proliferation of MPM cells.

### 3.4 Expression of IL7R and IL7 in MPM tumors

**Fig. 4:**
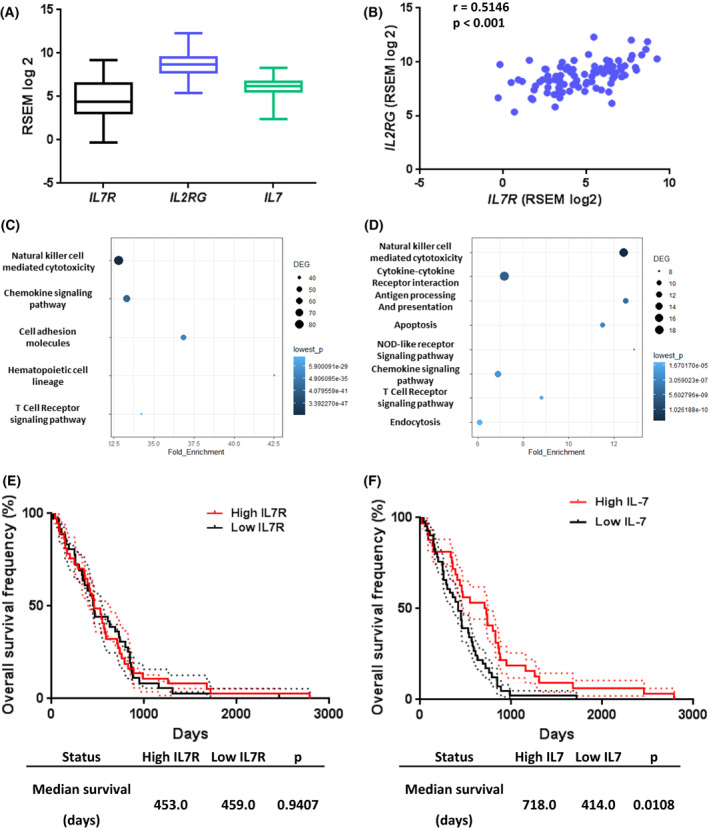
Expression of *IL7R* and *IL2RG* in MPM tumors (*n* = 87). RNASeq mRNA expression values were obtained from TCGA datasets. (A) mRNA expression of *IL7R*, *IL2RG* and *IL7* in MPM tumors. Graphs are whiskers plots (min to max). The line in the middle of the box is plotted at the median. (B) Correlation between *IL7R* and *IL2RG* mRNA expression in MPM tumors. Spearman test. (C, D) Pathway enrichment analyses based on genes positively associated with *IL7R* (C) and *IL7* (D) expression. (E, F) Patients were split into ‘high expression’ and ‘low expression’ groups based on the median of expression of *IL7R* (E) or *IL7* (F) mRNA expression in MPM tumors. Differences in survival between groups were assessed using log‐rank tests. Dotted lines represent standard error interval. MPM, malignant pleural mesothelioma.

*IL7R*, *IL2RG*, and *IL7* expressions in tumors were studied using the TCGA dataset. These two genes and *IL2RG* are expressed in MPM tumors (Fig. 4A). Expression of *IL7R* and *IL2RG* was strongly correlated (spearman *r* = 0.5146, *P* < 0.001) (Fig. 4B), whereas this correlation was weaker for *IL7R* and *IL7* (spearman *r* = 0.2678, *P* < 0.0122) (Fig. S7). Expression of *IL7R* is associated with expression of genes belonging to pathways involved in immune response such as cytokine–cytokine receptor interaction, chemokine signaling pathway, cell adhesion molecules, hematopoietic cell lineage, and T‐cell receptor signaling pathway (Fig. 4C). *IL7* expression was also associated with expression of genes belonging to pathways involved in immune response such as cytokine–cytokine receptor interaction, chemokine signaling pathway, cell adhesion molecules, hematopoietic cell lineage, and T‐cell receptor signaling pathway such as natural killer cell‐mediated cytotoxicity, cytokine–cytokine receptor interaction, antigen processing and presentation, apoptosis, NOD‐like receptor signaling pathway, chemokine signaling pathway, toll‐like receptor signaling pathway, and endocytosis (Fig. 4D). No association between *IL7R* expression and overall survival of patients was observed (Fig. 4E). However, we found that patients with high (above median) *IL7* gene expression in the tumors had better survival than those with low (below median) IL‐7 expression (median survival 795.0 days vs. 434 days; *P* = 0.0045) (Fig. 4F). This result seems independent on the MPM subtypes (Table 2) given that *IL7* expression was similar in epithelioid mesothelioma (EM), sarcomatoid mesothelioma (SM), biphasic mesothelioma (BM), and diffuse malignant mesothelioma (DM) (Fig. S8A) and that the prognostic value of *IL7* expression was also confirmed in EM patients (Fig. S8B). Moreover, univariate and multivariate analyses confirmed the independent association of *IL7* expression with overall survival of patients (Fig. S8C,D) even if histology remains the most significant factor associated with patient survival.

**Table 2:** Description of groups and demographic characteristics of recruited patients in TCGA.

	MPM
Description	74
	52 epithelioid
	3 sarcomatoid
	13 biphasic
	6 diffuse malignant mesothelioma
Age, years (mean ± SD)	62.88 ± 10.28
Male sex (%)	82

MPM, malignant pleural mesothelioma.

### 3.5 IL‐7 level in pleural effusions from MPM is associated with patient survival

**Fig. 5:**
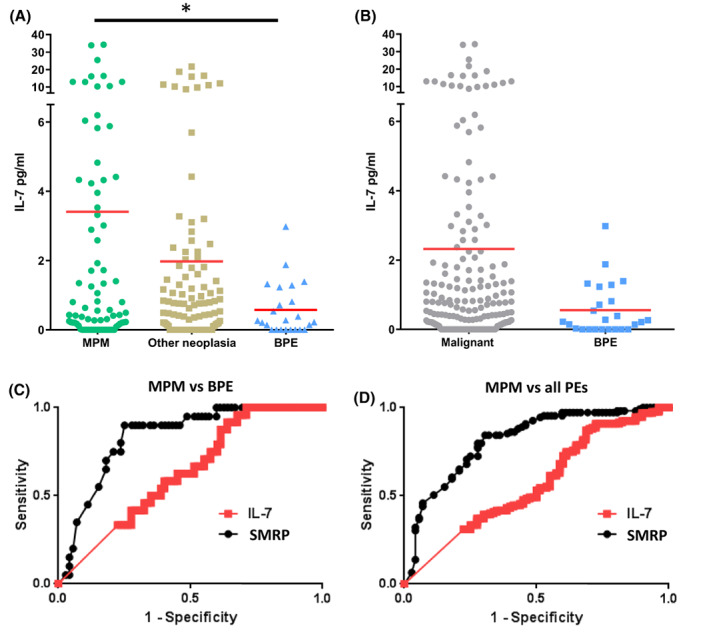
IL‐7 expression in pleural effusions from patients. IL‐7 and SMRP levels were measured in pleural effusions (PE) by ELISA. (A) Comparative expression of IL‐7 in PEs from MPM (*n* = 80), other neoplasia (*n* = 108), and BPE (*n* = 24). (B) Comparative expression of IL‐7 in malignant PE (*n* = 188) and BPE (*n* = 24). Red bars correspond to mean. (A, B) Mann–Whitney *t*‐test. *P < 0.05. (C, D) ROC curves for IL‐7 and SMRP to differentiate between MPM and BPE (C) or between MPM and all other PEs (other neoplasia + BPE) (D). BPE, benign pleural effusion; MPM, malignant pleural mesothelioma; ROC, receiver operating characteristic curve; SMRP, soluble mesothelin‐related peptide.

**Fig. 6:**
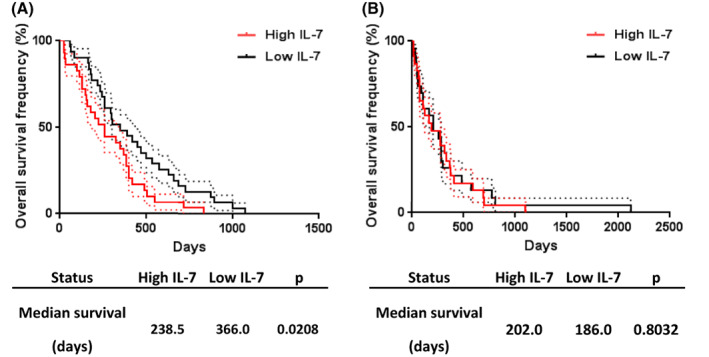
Prognostic value of IL‐7 expression in pleural effusions from patients with MPM (*n* = 65) or lung cancer (*n* = 46). Patients were split into ‘high expression’ and ‘low expression’ groups based on the median of expression of IL‐7 in MPM (A) or lung cancer (B) pleural effusions. Differences in survival between groups were assessed using log‐rank tests. Dotted lines represent standard error interval. MPM, malignant pleural mesothelioma.

We next measured IL‐7 concentration by ELISA in a collection of PEs from patients with MPM (*n* = 80), other neoplasia (*n* = 108) or benign pleural effusions (BPE) (*n* = 24) (Table 3 and Table S4). IL‐7 concentration was significantly higher in MPM PE (mean: 3.408 pg·mL^−1^) compared to BPE (mean: 0.5736 pg·mL^−1^) (*P* = 0.041) (Fig. 5A). No significant difference was observed between other neoplasia and MPM or BPE groups (Fig. 5A). When MPM and other neoplasia were combined into a malignant group (*n* = 188), IL‐7 levels still appeared to be higher in the malignant compared to the BPE group, but the difference was not significant (Fig. 5B). Among MPM patients, biphasic mesothelioma (BM) seemed to express low level of IL‐7 compared to sarcomatoid (SM) and epithelioid mesothelioma (EM) (Fig. S9). However, these observations should be confirmed in larger cohorts because of the low number of BM and SM samples in our biocollection. No differences were observed in the IL‐7 levels between MPM, lung cancer and other neoplasia PEs (Fig. S9). Because soluble mesothelin‐related protein (SMRP) is currently the best diagnostic biomarker for mesothelioma [20], we also measured SMRP concentration in PEs by ELISA and found a significant but weak correlation between IL‐7 and SMRP levels in MPM (Spearman *r* = 0.279; *P* = 0.0176) (Fig. S10A). We then evaluated the diagnostic value of pleural fluid IL‐7 using receiver operating characteristic (ROC) curve representation and area under the curve (AUC) determination (Fig. 5C,D and Table 4). Compared to SMRP, IL‐7 is a poor diagnostic biomarker to discriminate between MPM and BPE (AUC = 0.6313; *P* = 0.0519 for IL‐7; AUC = 0.8361; *P* < 0.0001 for SMRP) (Fig. 5C and Table 4) and between MPM and all other PEs (AUC = 0.5663; *P* = 0.1059 for IL‐7; AUC = 0.8166; *P* < 0.0001 for SMRP) (Fig. 5D and Table 4). Next, we evaluated the prognostic value of IL‐7 concentration in PE of patients with MPM. Overall survival data were available for 65 patients and global median survival for patients with MPM was 300.5 days. Patients were separated in two groups according to the IL‐7 median (Fig. 6A). Patients with IL‐7 levels above the median presented a significant lower survival than those with IL‐7 below the median (238.5 days vs. 366.0 days; *P* = 0.0208; Hazard Ratio = 1.53) (Fig. 6A). When only patients with epithelioid MPM (*n* = 55) were analyzed, patients with high IL‐7 levels still had lower survival compared to those with low IL‐7 levels (median survival 202.0 days vs. 366.0 days; *P* = 0.0373; hazard ratio = 1.90) (Fig. S11). This independent association of IL‐7 with overall survival of patients was confirmed using univariate and multivariate analyses (Fig. S11C,D). SMRP levels in PE, however, were not associated with MPM patient survival (Fig. S10B). We asked whether pleural fluid IL‐7 also had prognostic value in PE secondary to lung cancer. We did the same analyses in a subgroup of 46 patients with PE secondary to lung cancer for whom follow‐up data were available and found that IL‐7 levels were not associated with patient survival (Fig. 6B). Finally, we also measured IL‐7 in the serum of patients with MPM and BPE by ELISA and found no difference in the IL‐7 levels between these two groups, the mean values were 15.775 and 15 778 pg·mL^−1^ for MPM and BPE, respectively (Fig. S12).

**Table 3:** Description of groups and demographic characteristics of recruited patients for pleural effusion study.

	MPM	Other neoplasia	BPE
Description	80	108	24
	63 epithelioid	62 Lung	
	6 sarcomatoid	44 others	
	6 biphasic		
	5 unspecified		
Age, years (mean ± SD)	69.03 ± 9.66	64.39 ± 12.72	73.17 ± 11.30
Male sex (%)	83.7	50.0	87.5
Confirmed asbestos exposure (%)	68.7	12.9	33.3

MPM, malignant pleural mesothelioma; BPE, benign pleural effusion.

**Table 4:** ROC curve data for ability of IL‐7 and SMRP to differentiate MPM from ADCA and/or BPE in pleural effusions.

	AUC	95% confidence interval	SE	*P*
MPM vs BPE				
SMRP	0.8361	0.7490 to 0.9233	0.044	< 0.0001
IL‐7	0.6313	0.5176 to 0.7449	0.057	0.0519
MPM vs all PE				
SMRP	0.8166	0.7517 to 0.8816	0.033	< 0.0001
IL‐7	0.5663	0.4851 to 0.6475	0.041	0.1059
EM vs all PE				
SMRP	0.8152	0.7545 to 0.8758	0.030	< 0.0001
IL‐7	0.6058	0.5184 to 0.6931	0.044	0.0171

AUC, area under the curve; BPE, benign pleural effusion; EM, epithelioid mesothelioma; MPM, malignant pleural mesothelioma; PE, pleural effusion; ROC, receiver operating characteristic; SE, standard error.

## 4 Discussion

In this study, we aimed at evaluating the IL‐7R/IL‐7 pathway in malignant pleural mesothelioma (MPM). We found that CD127 and IL‐7 are expressed by a majority of MPM cells. We observed that expression of CD127 is not always associated with expression of CD132 and in the cell lines which expressed complete IL‐7R, the IL‐7 signaling pathway is not functional. We also analyzed the TCGA dataset and confirmed the expression of *IL7R* and *IL7* in MPM tumors. *IL7R* expression is strongly associated with expression of genes involved in immune response. Interestingly, tumor expression of *IL7* was not only associated with pathways involved in immune response but with increased overall survival of patients. Finally, the study of the expression of IL‐7 in pleural effusions (PEs) showed a significant higher level of IL‐7 in MPM PE compared to benign PE and that high IL‐7 level in the PE was associated with lower survival of patients in MPM but not in lung cancer patients.

In a previous study using immunohistology, it was shown that high IL‐7R expression in MPM tumor was associated with worse outcomes [4]. Here, we observed that more than 50% of our cell lines expressed IL‐7R (CD127). However, our analyses of the TCGA dataset did not give evidence of a correlation between *IL7R* gene expression and patient survival. This difference can be explained by the two different methods used to quantify IL‐7R expression. In the TCGA dataset, IL‐7R gene expression was measured by RNASeq and represents the sum of both tumor cells and infiltrating cell expression, whereas the immunohistological method evaluated IL‐7R protein expression on tumor cells.

At the mRNA level, our analyses of the TCGA dataset showed that high *IL7* expression in MPM tumors was associated with increased overall patient survival. The prognostic value of *IL7* expression seemed independent of the MPM subtypes. Pathway analyses showed that in *IL7*‐expressing tumors, there was an enrichment of genes involved in immune response, which may partially explain the increased patient survival. Since tumor‐infiltrating immune cells may also express IL‐7R, it is surprising that we did not find a correlation between tumor IL‐7R gene expression and patient survival. Additional correlation analyses showed that *IL7R* expression was significantly associated with markers of cells involved in immunosuppression such as *CD68*, *CD163*, *MRC1*, and *IL10* (tumor‐associated macrophages), *CD4*, *FOXP3* (regulator T lymphocytes) and *CD274* (PD‐L1) (Table S5). Those genes were not found to be associated with *IL7* expression except *CD274* and *CD8a* but with a low Spearman *R* value (0.270 and 0.272, respectively) (Table S6). These data suggest that *IL7* expression in tumors could be associated to a better immune response, whereas *IL7R*‐expressing tumors were not.

It has been shown that high serum IL‐7 levels may help to distinguish patients with prostate cancer from those with benign prostate hypertrophy [21], unfortunately, it does not seem to be the case in our study, serum IL‐7 could not distinguish MPM from BPE. However, in PE, we found that IL‐7 concentrations were significantly higher in MPM patients compared to patients with BPE (Fig. 5A) but PE IL‐7 was less potent than PE SMRP in distinguishing MPM from BPE (Fig. 5C). However, our results suggest that pleural fluid IL‐7 may be a potential prognostic biomarker because it was associated with lower overall patient survival. High expression of IL‐7 was already shown to be associated with lower survival of patients with other cancers such as lung cancer [14] or breast cancer [15].

The reason why high mRNA expression of IL‐7 in MPM tumors was associated with better patient survival, whereas high IL‐7 protein levels in PE were associated with shorter survival is not clear. First of all, there may be a discrepancy between mRNA and protein expressions. Secondly, the two techniques measure IL‐7 expression in two different manners, whereas RNASeq measures the number of IL‐7 mRNA copies present in the tumor at a single time point, the time of biopsy, IL‐7 levels in PE result from the accumulation over time of this cytokine in the PE. Finally, RNASeq data are the reflection of the local *IL7* mRNA expression in the tumor, including tumor and infiltrated cells (immune, stromal, and endothelial cells among others), whereas in PE, concentrations measured are the reflection of IL‐7 secretion from the pleural cavity including secretion from tumor, immune cell infiltrate and healthy pleural tissue. In addition, disparate findings have been observed for other prognostic biomarkers such as chemerin, an adipokine involved in inflammation. Indeed, in patients with nonsmall cell lung cancer (NSCLC), high serum chemerin levels were shown to be associated with shorter overall survival [22], whereas high chemerin expression in tumor as assessed by immunohistology was found to be associated with better survival [23]. Finally, our study was performed on a relatively small number of patients, larger cohorts are needed to confirm these findings.

In PEs, the association of high IL‐7 levels with lower survival of patients could be related to the tumor development. Since MPM cells can secrete IL‐7, as shown for some other malignant cells [24, 25, 26], the higher IL‐7 levels in PE may reflect higher tumor burden and serve as a prognostic biomarker of tumor development. Moreover, several publications have described an autocrine action of the IL‐7/IL‐7R pathway on tumor cells to promote cell proliferation [19]. However, in MPM, it seems this was not the case. Indeed, although some cell lines expressed IL‐7R (CD127/CD132) and secrete IL‐7, no constitutive activation and IL‐7‐induced STAT5 phosphorylation and proliferation were observed. Compared to PBMCs, MPM cell lines only weakly expressed molecules of the JAK–STAT pathway, which could explain the absence of IL‐7 effect. Finally, this signaling pathway seems to be not involved in mesothelioma cell proliferation but may contribute to fuel inflammation, by acting on other cells, and thus promote tumor development, as it was previously suggested for other cancers [27, 28]. Indeed, the blocking of IL‐7R, in a mice model of melanoma, suppressed tumor growth by modifying immune infiltrate suggesting that IL‐7/IL‐7R axis could be an interesting target for therapy. This approach could be combined with current immunotherapy to improve their antitumor effect.

## 5 Conclusion

In conclusion, this study demonstrates that IL‐7R is expressed by around 50% of MPM cell lines and, despite expression of CD132 in most cases, that this receptor is not only functional but also that the expression of IL‐7 in MPM PEs, in particular, could have a prognostic value and constitute a new therapeutic target. However, for these last points, validation on independent cohorts and additional studies are needed to confirm our conclusions.

## Conflict of interest

The authors declare no conflict of interest.

## Author contributions

HL‐M, SB, and CB performed experimental study design and wrote the manuscript. AL‐C provided pleural fluids. HL‐M, SD, TVH‐N, and VD performed the experiments. HL‐M, AL‐C, SB, and CB analyzed and interpreted the data. All authors reviewed the manuscript.

### Peer review

The peer review history for this article is available at https://publons.com/publon/10.1002/1878‐0261.13310.

## Supporting information


**Fig. S1.** Correlation between *IL7* mRNA expression and secretion in MPM cells.

**Fig. S2.** Correlation between *IL7* and *IL7R* mRNA expression in MPM cells.

**Fig. S3.** Characteristic of cell lines used in fig. 4.

**Fig. S4.** Expression of genes involved in IL‐7 signaling in PBMC.

**Fig. S5.** Expression of STAT5 and JAK1 in MPM cells.

**Fig. S6.** Evaluation of STAT5 phosphorylation 5 (Y694) following IL‐7 treatment in MPM cells.

**Fig. S7.** Correlation between IL7 and IL7R mRNA expression in MPM tumors.

**Fig. S8.** Expression and prognostic value of IL7 gene in MPM subtype.

**Fig. S9.** Expression of IL‐7 in pleural effusions of subgroup of patients.

**Fig. S10.** Correlation between IL‐7 and SMRP expression and prognostic value of SMRP in pleural effusions from MPM patients.

**Fig. S11.** Prognostic value of IL‐7 expression in pleural effusions from patients with epithelioid MPM.

**Fig. S12.** Expression of IL‐7 in serum from patients with MPM or BPE.

**Table S1.** Characteristics of the patients from which the cell lines were established.

**Table S2.** Correlation between *IL7R* and *IL7*, *IL2R*, *TSLP*, and *TSLPR* gene expression in MPM cell lines.

**Table S3.** Sensitivity of MPM cell lines to JAK1 inhibitor ruxolitinib.

**Table S4.** Description of groups and demographic characteristics of recruited patients for pleural effusion study.

**Table S5.** Correlation of IL7R expression with markers of immune cells in TCGA database.

**Table S6.** Correlation of IL7 expression with markers of immune cells in TCGA database.

## Data Availability

The datasets used and analyzed during the current study are available from the corresponding author.
