# Supplementary material for: IL‐7 is expressed in malignant mesothelioma and has a prognostic value
Source: Mol Oncol. 2022 Sep 10;16(20):3606–19. doi: 10.1002/1878-0261.13310 (PMC9580880; doi:10.1002/1878-0261.13310)
Supplement: Supplementary file 1 — Fig. S1. Correlation between IL7 mRNA expression and secretion in MPM cells. [file MOL2-16-3606-s014.pdf]

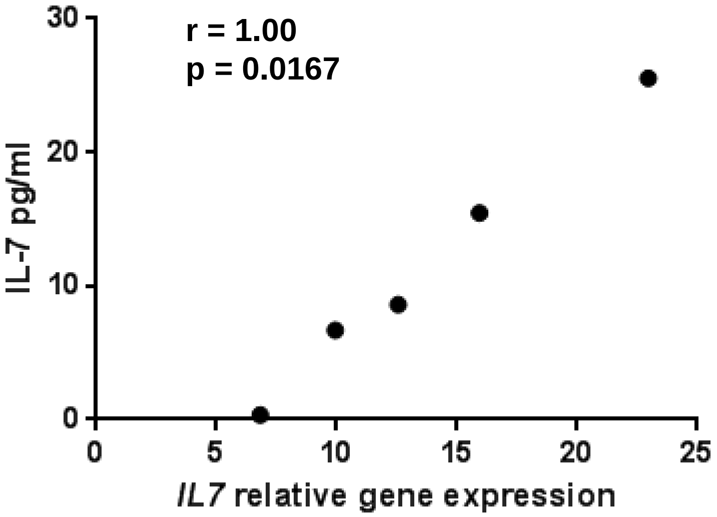

Supplementary figure 1: Correlation between *IL7* mRNA expression and secretion in MPM cells. mRNA expression was measured using RT-PCR and IL-7 secretion was determined using ELISA assay. MPM, malignant pleural mesothelioma.
