# Supplementary material for: IL‐7 is expressed in malignant mesothelioma and has a prognostic value
Source: Mol Oncol. 2022 Sep 10;16(20):3606–19. doi: 10.1002/1878-0261.13310 (PMC9580880; doi:10.1002/1878-0261.13310)
Supplement: Supplementary file 3 — Fig. S3. Characteristic of cell lines used in fig. 4. [file MOL2-16-3606-s001.pdf]

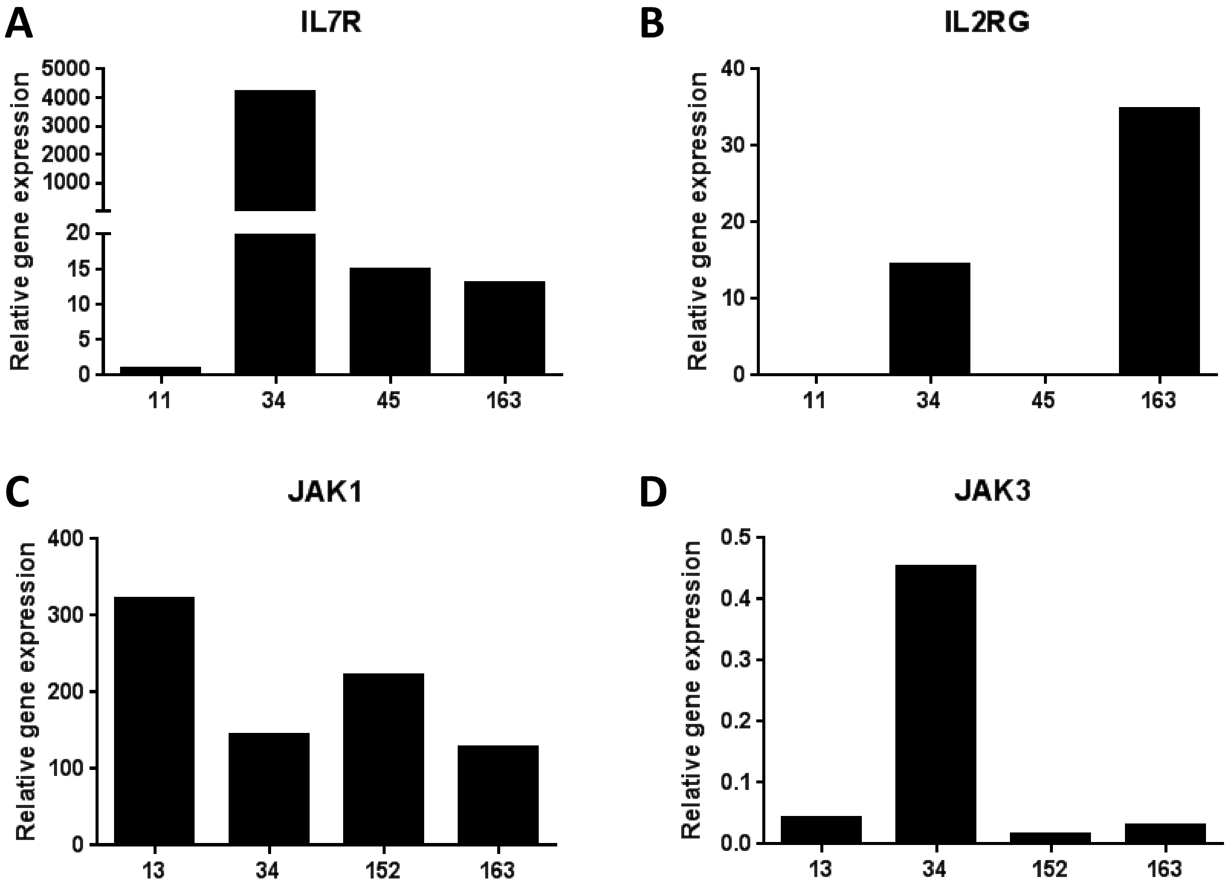

Supplementary figure 3: Characteristic of cell lines used in figure 3. *IL7R* (A), *IL2R* (B), *JAK1* (C) and *JAK3* (D) gene expressions were determined using RT-PCR in 4 MPM cell lines Meso 11, 34, 45, and 163. MPM, malignant pleural mesothelioma.
