# Supplementary material for: IL‐7 is expressed in malignant mesothelioma and has a prognostic value
Source: Mol Oncol. 2022 Sep 10;16(20):3606–19. doi: 10.1002/1878-0261.13310 (PMC9580880; doi:10.1002/1878-0261.13310)
Supplement: Supplementary file 4 — Fig. S4. Expression of genes involved in IL‐7 signaling in PBMC. [file MOL2-16-3606-s009.pdf]

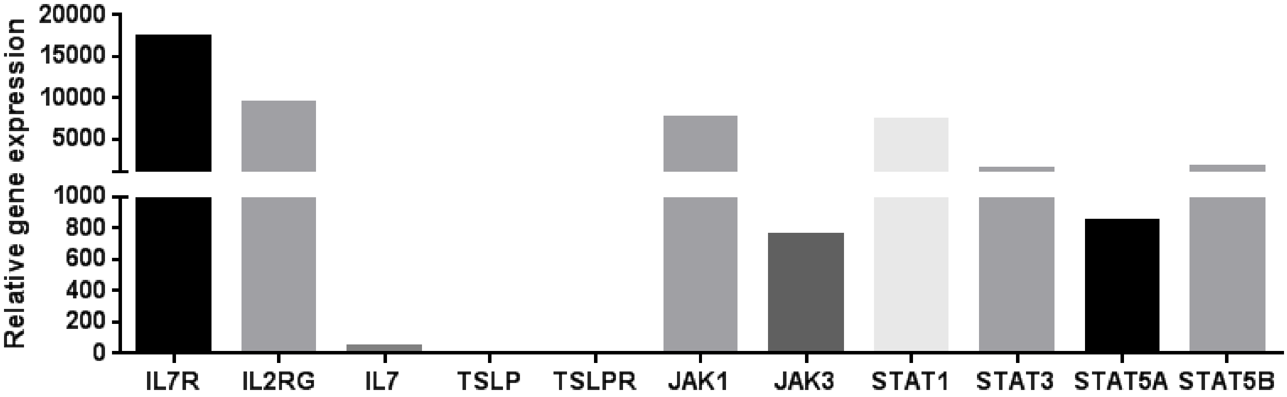

Supplementary figure 4: Expression of genes involved in IL-7 signalling in PBMC. PBMC were obtained from blood of healthy donor and isolated using Ficoll gradient. Gene mRNA expression was measured using RT-PCR. PBMC, peripheral blood mononuclear cells.
