# Supplementary material for: IL‐7 is expressed in malignant mesothelioma and has a prognostic value
Source: Mol Oncol. 2022 Sep 10;16(20):3606–19. doi: 10.1002/1878-0261.13310 (PMC9580880; doi:10.1002/1878-0261.13310)
Supplement: Supplementary file 5 — Fig. S5. Expression of STAT5 and JAK1 in MPM cells. [file MOL2-16-3606-s017.pdf]

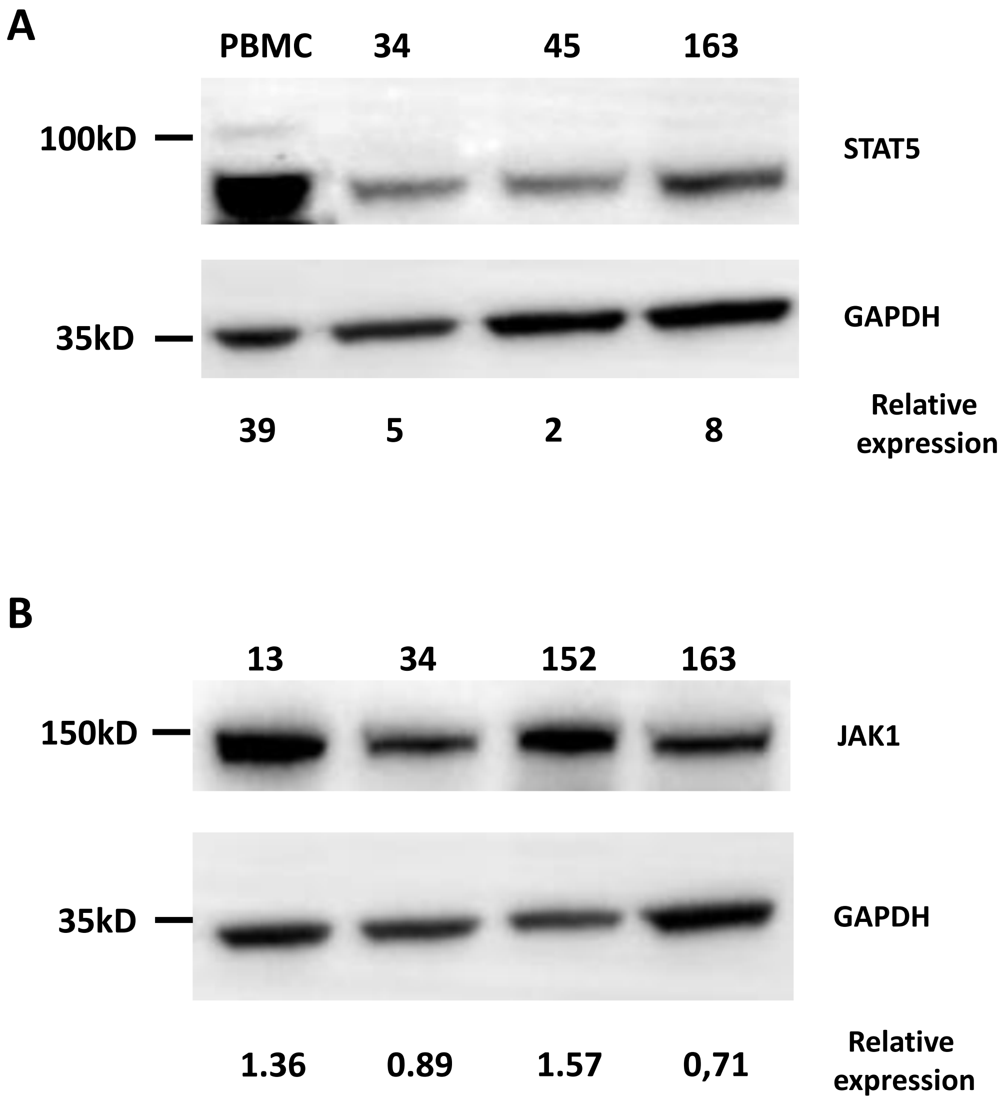

Supplementary figure 5: Expression of STAT5 (A) and JAK1 (B) in MPM cells. Cells were lysed in RIPA buffer containing a Protease Inhibitor Cocktail (Sigma) and denatured at 95°C for 5 minutes in Laemmli buffer with 10% b-mercaptoethanol. Then, 10 µg of proteins for cellular lysate were separated by sodium dodecyl sulfate polyacrylamide gel electrophoresis on 8% gels and transferred to polyvinylidene difluoride membranes. Blots were incubated with anti-STAT5 (Clone 89, BD Biosciences), anti-JAK1 (Clone 73/JAK1, BD Biosciences) or anti-GAPDH (Clone 47, BD Biosciences) as control at 0.5 µg/ml in PBS 5 % milk followed by incubation with HRP-coupled secondary antibodies (Jackson Immunoresearch). Proteins were revealed using Enhanced Chemiluminescence Detection ECL (Bio-Rad). Relative expression corresponds to the ratio of the JAK1 signal intensity to the GAPDH signal intensity. Quantifications were performed using Image J-Win64 software. MPM, malignant pleural mesothelioma; PBMC, peripheral blood mononuclear cells.
