# Supplementary material for: IL‐7 is expressed in malignant mesothelioma and has a prognostic value
Source: Mol Oncol. 2022 Sep 10;16(20):3606–19. doi: 10.1002/1878-0261.13310 (PMC9580880; doi:10.1002/1878-0261.13310)
Supplement: Supplementary file 6 — Fig. S6. Evaluation of STAT5 phosphorylation 5 (Y694) following IL‐7 treatment in MPM cells. [file MOL2-16-3606-s004.pdf]

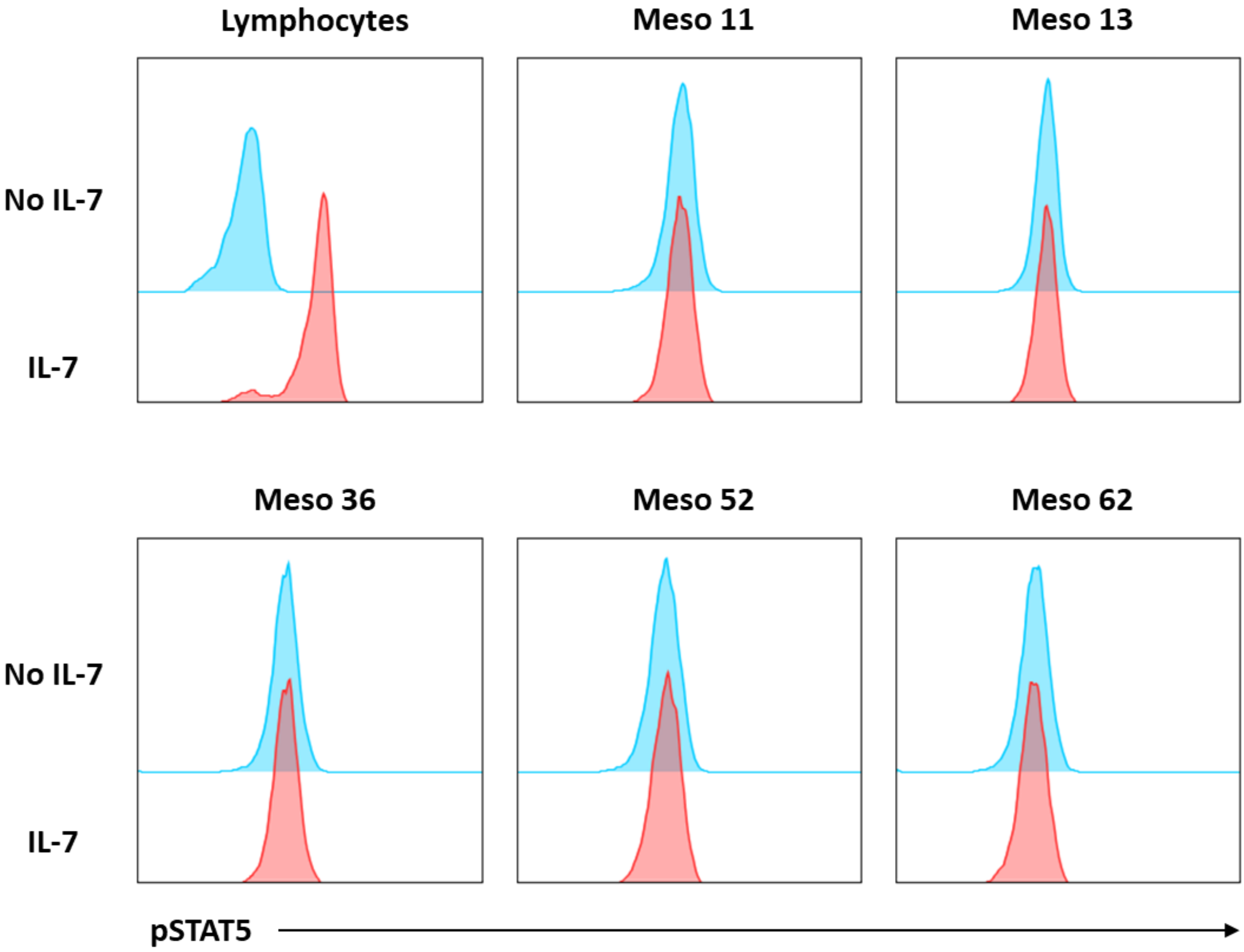

Supplementary figure 6: Evaluation of STAT5 phosphorylation 5 (Y694) following IL-7 treatment in MPM cells. PBMCs, obtained from blood of healthy donor and isolated using Ficoll gradient, and MPM cells were incubated for 15 minutes with 10 ng/ml of IL-7. Then, p(Y694)STAT5 was measured using flow cytometry. MPM, malignant pleural mesothelioma; PBMC, peripheral blood mononuclear cells.
