# Supplementary material for: IL‐7 is expressed in malignant mesothelioma and has a prognostic value
Source: Mol Oncol. 2022 Sep 10;16(20):3606–19. doi: 10.1002/1878-0261.13310 (PMC9580880; doi:10.1002/1878-0261.13310)
Supplement: Supplementary file 7 — Fig. S7. Correlation between IL7 and IL7R mRNA expression in MPM tumors. [file MOL2-16-3606-s007.pdf]

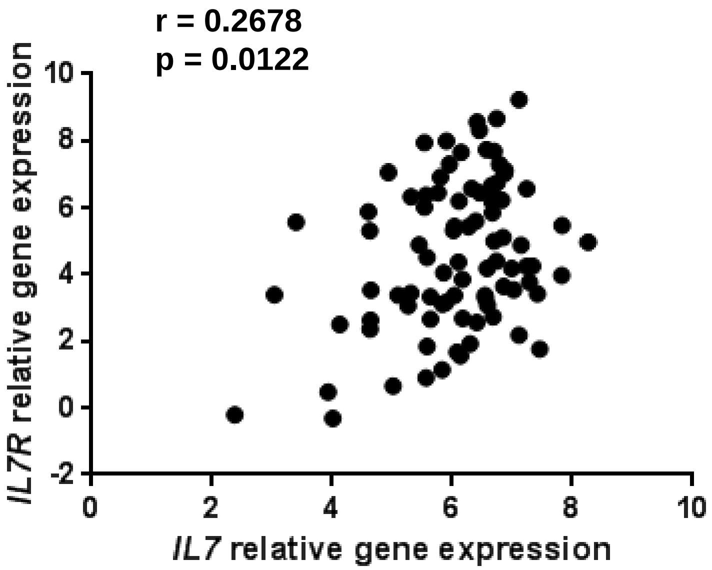

Supplementary figure 7: Correlation between *IL7* and *IL7R* mRNA expression in MPM tumours. RNASeq mRNA expression values were obtained from TCGA datasets. Correlation was analyzed using non-parametric Spearman test. MPM, malignant pleural mesothelioma; TCGA, the cancer genome atlas.
