# Supplementary material for: IL‐7 is expressed in malignant mesothelioma and has a prognostic value
Source: Mol Oncol. 2022 Sep 10;16(20):3606–19. doi: 10.1002/1878-0261.13310 (PMC9580880; doi:10.1002/1878-0261.13310)
Supplement: Supplementary file 8 — Fig. S8. Expression and prognostic value of IL7 gene in MPM subtype. [file MOL2-16-3606-s002.pdf]

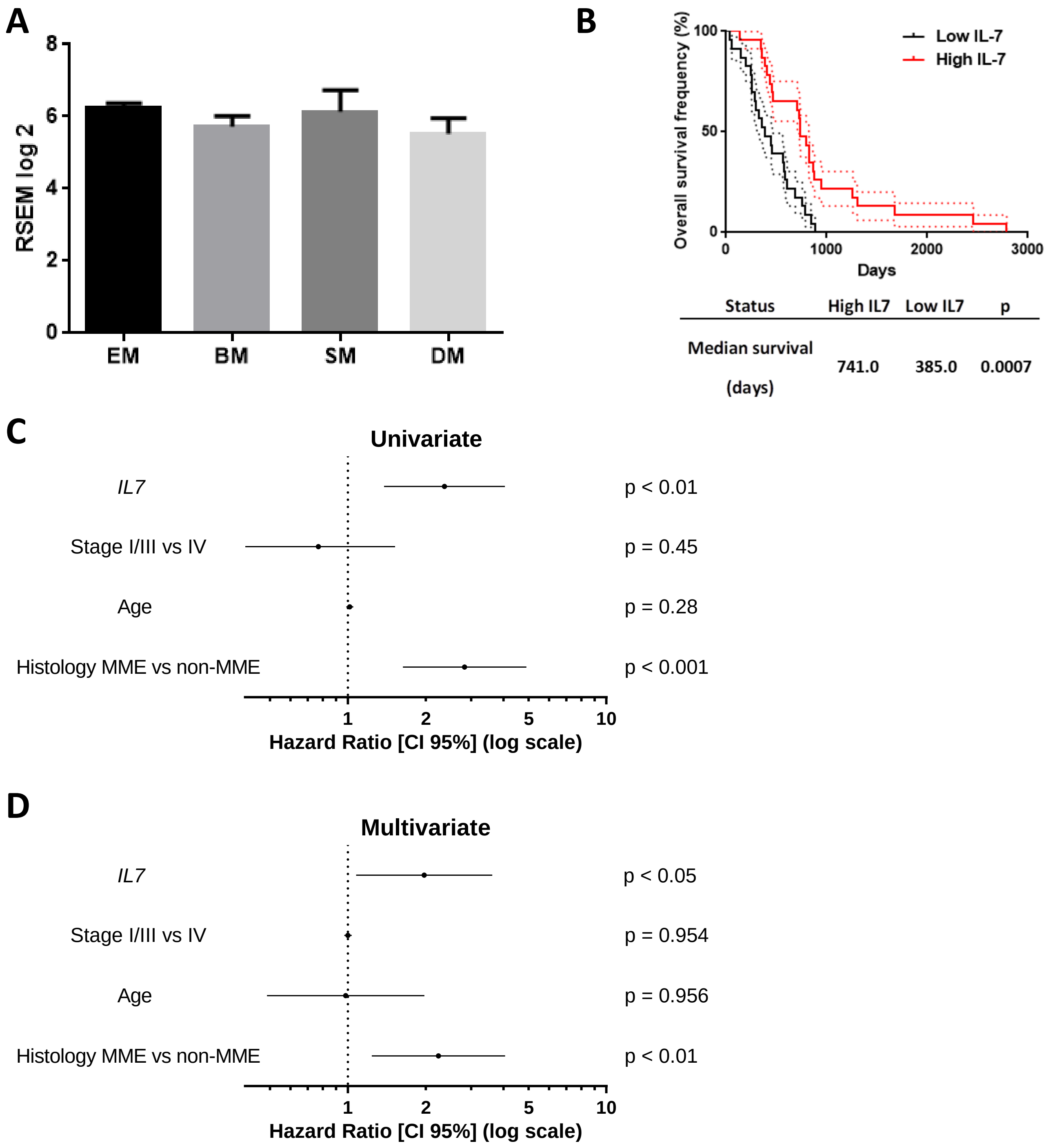

Supplementary figure 8: Expression and prognostic value of *IL7* gene in MPM subtype. RNASeq mRNA expression values were obtained from TCGA datasets. **A**, mRNA expression of *IL7* in MPM subtypes. **B**, epithelioid mesothelioma patients were split in 'high expression' and 'low expression' groups based on the median of expression of *IL7* mRNA expression in MPM tumours. Differences in survival between groups were assessed using log-rank tests. **C**,**D)** Univariate (C) and multivariate (D) Cox regression analysis of overall survival in MPM patients. Forest plots show hazard ratios (HR) and 95% confidence interval (CI) for overall survival according to expression, age at diagnostic, tumor stage and histology based on a threshold of 0.22. For *IL7* expression, samples were discriminated on the basis of the median expressionor the absence of at least one mutation in one of the genes. For histology, MMB, MMS, and MMD were classified as non_MME. MPM, malignant pleural mesothelioma; TCGA, the cancer genome atlas. MME, epithelioid MPM; MMB, biphasic MPM; MMS, sarcomatoid MPM; MMD, desmoplastic MPM.
