# Supplementary material for: IL‐7 is expressed in malignant mesothelioma and has a prognostic value
Source: Mol Oncol. 2022 Sep 10;16(20):3606–19. doi: 10.1002/1878-0261.13310 (PMC9580880; doi:10.1002/1878-0261.13310)
Supplement: Supplementary file 9 — Fig. S9. Expression of IL‐7 in pleural effusions of subgroup of patients. [file MOL2-16-3606-s013.pdf]

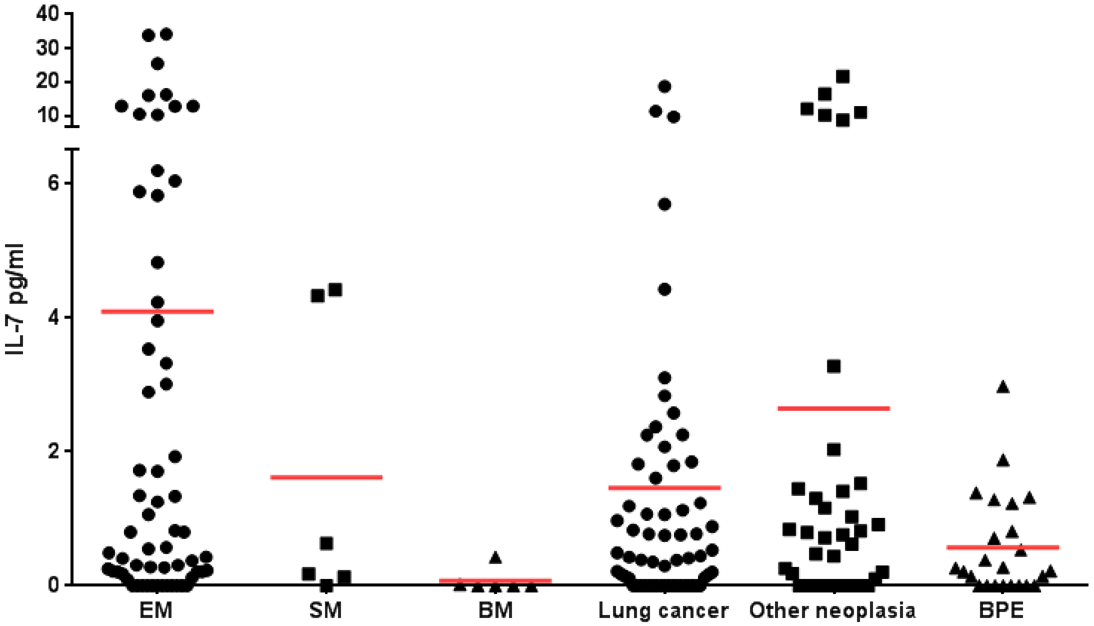

Supplementary figure 9: Expression of IL-7 in pleural effusions of subgroups of patients. IL-7 was measured using ELISA assay. Red bars correspond to mean values. EM, epithelioid mesothelioma; SM, sarcomatoid mesothelioma; BM, biphasic mesothelioma; BPE, benign pleural effusion.
