# Supplementary material for: IL‐7 is expressed in malignant mesothelioma and has a prognostic value
Source: Mol Oncol. 2022 Sep 10;16(20):3606–19. doi: 10.1002/1878-0261.13310 (PMC9580880; doi:10.1002/1878-0261.13310)
Supplement: Supplementary file 10 — Fig. S10. Correlation between IL‐7 and SMRP expression and prognostic value of SMRP in pleural effusions from MPM patients. [file MOL2-16-3606-s018.pdf]

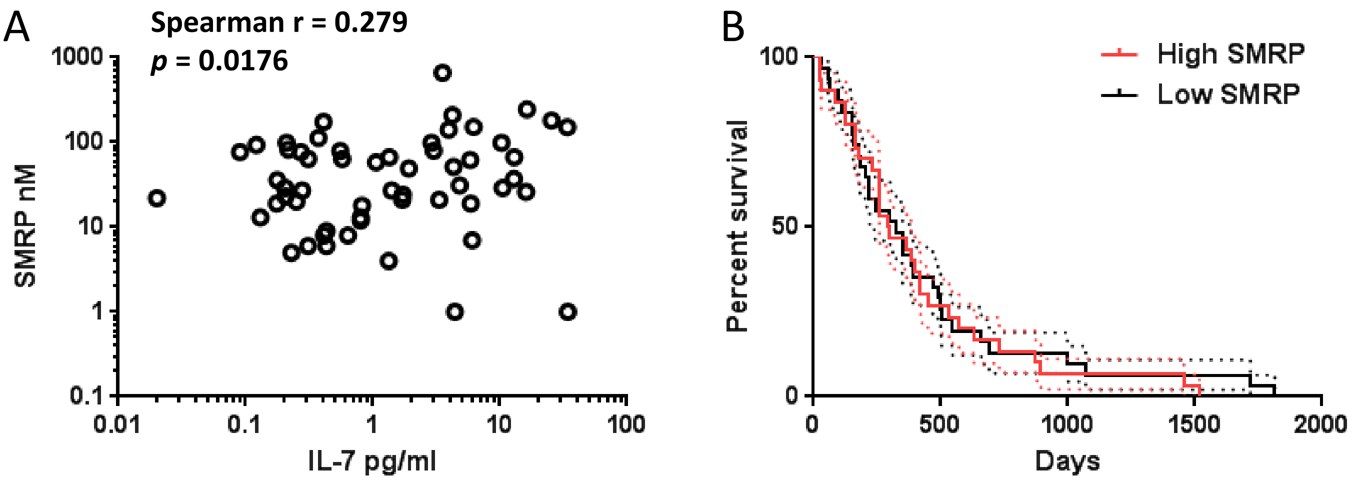

Supplementary figure 10: Correlation between IL-7 and SMRP expression and prognostic value of SMRP in pleural effusions from MPM patients. IL-7 and SMRP were measured using ELISA assay. A) Correlation between the expression of IL-7 and SMRP in MPM PEs. B) Patients were split in ‘high expression’ and ‘low expression’ groups based on the median of expression of SMRP in MPM PEs. Differences in survival between groups were assessed using log-rank tests. Dotted lines represent standard error interval. MPM, malignant pleural mesothelioma; SMRP, soluble mesothelin-related peptide.
