# Supplementary material for: IL‐7 is expressed in malignant mesothelioma and has a prognostic value
Source: Mol Oncol. 2022 Sep 10;16(20):3606–19. doi: 10.1002/1878-0261.13310 (PMC9580880; doi:10.1002/1878-0261.13310)
Supplement: Supplementary file 11 — Fig. S11. Prognostic value of IL‐7 expression in pleural effusions from patients with epithelioid MPM. [file MOL2-16-3606-s005.pdf]

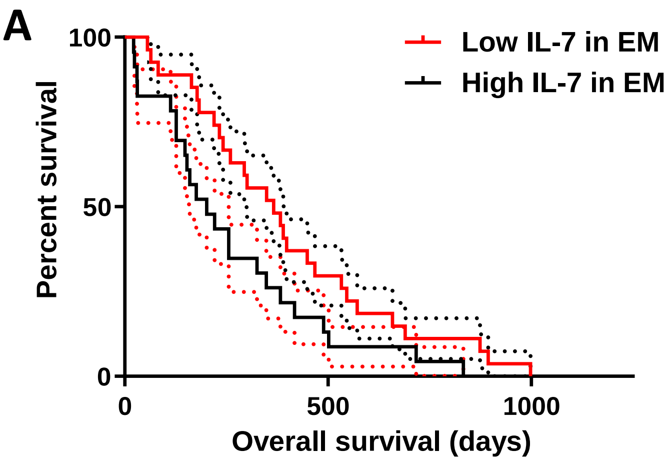

Status	High IL7	Low IL7	p
Median survival (days)	202.0	366.0	0.0373

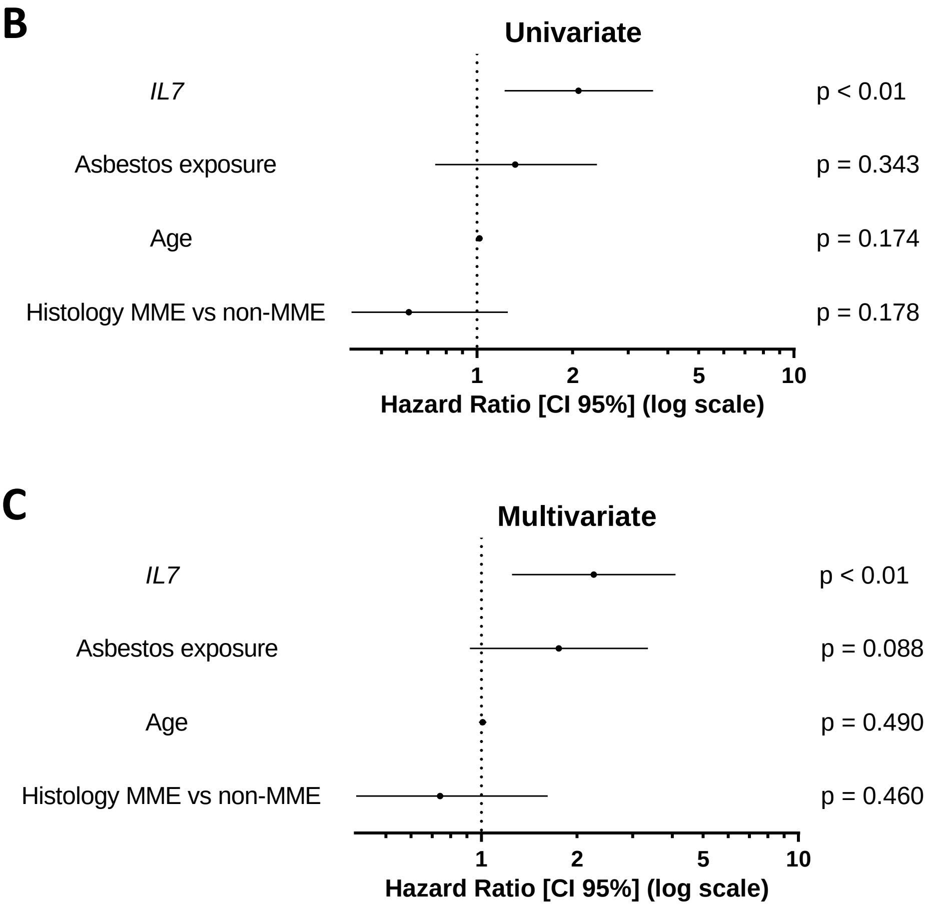

Supplementary figure 11: Prognostic value of IL-7 expression in pleural effusions from patients with epithelioid MPM. A) Patients were split in 'high expression' and 'low expression' groups based on the median of expression of IL-7 in epithelioid MPM pleural effusions. Differences in survival between groups were assessed using log-rank tests. Dotted lines represent standard error interval. **B, C**) Univariate (B) and multivariate (C) Cox regression analysis of overall survival in MPM patients. Forest plots show hazard ratios (HR) and 95% confidence interval (CI) for overall survival according to IL-7 expression, asbestos exposure, age at diagnostic, and histology based on a threshold of 0.22. For IL-7 expression, samples were discriminated on the basis of the median expression. For histology, MMB, MMS, and MMD were classified as non_MME. MPM, malignant pleural mesothelioma; TCGA, the cancer genome atlas. MME, epithelioid MPM; MMB, biphasic MPM; MMS, sarcomatoid MPM; MMD, desmoplastic MPM.
