# Supplementary material for: IL‐7 is expressed in malignant mesothelioma and has a prognostic value
Source: Mol Oncol. 2022 Sep 10;16(20):3606–19. doi: 10.1002/1878-0261.13310 (PMC9580880; doi:10.1002/1878-0261.13310)
Supplement: Supplementary file 12 — Fig. S12. Expression of IL‐7 in serum from patients with MPM or BPE. [file MOL2-16-3606-s012.pdf]

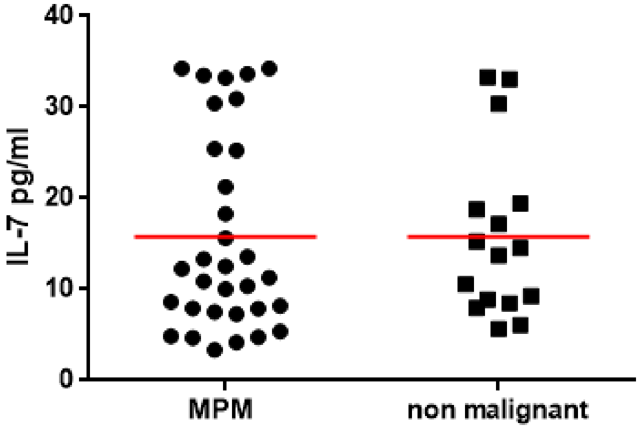

Supplementary figure 12: Expression of IL-7 in serum from patients with MPM or BPE. IL-7 was measured using ELISA. Red bars correspond to mean values. MPM, malignant pleural mesothelioma; BPE, benign pleural effusion.
