# Supplementary material for: IL‐7 is expressed in malignant mesothelioma and has a prognostic value
Source: Mol Oncol. 2022 Sep 10;16(20):3606–19. doi: 10.1002/1878-0261.13310 (PMC9580880; doi:10.1002/1878-0261.13310)
Supplement: Supplementary file 13 — Table S1. Characteristics of the patients from which the cell lines were established. [file MOL2-16-3606-s003.docx]

| Cell lines | Sex | Age | Pathology |
| --- | --- | --- | --- |
| Meso 4 | M | 60 | EM |
| Meso 11 | M | 79 | EM |
| Meso 13 | M | 81 | EM |
| Meso 34 | F | 77 | EM |
| Meso 35 | M | 53 | EM |
| Meso 36 | M | 74 | EM |
| Meso 37 | M | 66 | EM |
| Meso 45 | M | 73 | EM |
| Meso 47 | M | 84 | EM |
| Meso 52 | F | 65 | EM |
| Meso 56 | M | 79 | EM |
| Meso 61 | F | 66 | EM |
| Meso 62 | M | 78 | BM |
| Meso 76 | M | 72 | EM |
| Meso 96 | M | 68 | EM |
| Meso 122 | M | 73 | EM |
| Meso 144 | M | 58 | EM |
| Meso 148 | M | 74 | EM |
| Meso 150 | M | 59 | EM |
| Meso 152 | M | 64 | SM |
| Meso 163 | F | 63 | MPM |
| Meso 182 | M | 86 | MPM |
| ADCA 3 | M | 64 | Lung adenocarcinoma |
| ADCA 72 | M | 60 | Lung adenocarcinoma |
| ADCA 115 | F | 70 | Lung adenocarcinoma |
| ADCA 117 | M | 86 | Lung adenocarcinoma |
| ADCA 153 | M | 35 | Lung adenocarcinoma |
| ADCA 175 | M | 63 | Lung adenocarcinoma |
| A549 | M | 58 | Lung carcinoma |

Table S1 : Characteristic of the patients from which the cell lines were established.

MPM, malignant pleural mesothelioma ; EM, epithelioid mesothelioma, BM, biphasic mesothelioma ; SM, sarcomatoid mesothelioma.
