# Supplementary material for: IL‐7 is expressed in malignant mesothelioma and has a prognostic value
Source: Mol Oncol. 2022 Sep 10;16(20):3606–19. doi: 10.1002/1878-0261.13310 (PMC9580880; doi:10.1002/1878-0261.13310)
Supplement: Supplementary file 14 — Table S2. Correlation between IL7R and IL7, IL2R, TSLP, and TSLPR gene expression in MPM cell lines. [file MOL2-16-3606-s008.docx]

Table S2: Correlation between *IL7R* and *IL7*, *IL2R*, *TSLP* and *TSLPR* gene expression in MPM cell lines.

| *IL7R vs* | *IL2RG* | *IL7* | *TSLP* | *TSLPR* |
| --- | --- | --- | --- | --- |
| Spearman r | 0.779 | 0.398 | 0.0321 | 0.567 |
| 95% confidence interval | 0.524 to 0.906 | -0.0415 to 0.708 | -0.405 to 0.458 | 0.178 to 0.802 |
| p value | < 0.0001 | 0.0665 | 0.8869 | 0.0059 |
