# Supplementary material for: IL‐7 is expressed in malignant mesothelioma and has a prognostic value
Source: Mol Oncol. 2022 Sep 10;16(20):3606–19. doi: 10.1002/1878-0261.13310 (PMC9580880; doi:10.1002/1878-0261.13310)
Supplement: Supplementary file 15 — Table S3. Sensitivity of MPM cell lines to JAK1 inhibitor ruxolitinib. [file MOL2-16-3606-s015.docx]

Table S3: Sensitivity of MPM cell lines to JAK1 inhibitors Ruxolitinib

|  | Meso 13 | Meso 34 | Meso 152 | Meso 163 |
| --- | --- | --- | --- | --- |
| Area under the curve | 2247 | 1847 | 1580 | 1896 |
| *JAK1* relative  mRNA expression | 321.4 | 144.1 | 223 | 128.1 |
