# Supplementary material for: IL‐7 is expressed in malignant mesothelioma and has a prognostic value
Source: Mol Oncol. 2022 Sep 10;16(20):3606–19. doi: 10.1002/1878-0261.13310 (PMC9580880; doi:10.1002/1878-0261.13310)
Supplement: Supplementary file 16 — Table S4. Description of groups and demographic characteristics of recruited patients for pleural effusion study. [file MOL2-16-3606-s006.docx]

Table S4: Description of groups and demographic characteristics of recruited patients for pleural effusion study

|  | **Other neoplasia** | **BPE** |
| --- | --- | --- |
| Description | 108  62 Lung  10 breast  4 ovary  3 colon  3 leukemia  2 melanoma  1 lymphoma  1 pancreas  1 rectum  21 others | 24  20 Fibrosis+ pleural thickening  1 Rheumatoid arthritis  1 surgery  1 drug-related  1 Transudate |
| Age, y (mean ± SD) | 64.39 ± 12.72 | 73.17 ± 11.30 |
| Male sex, (%) | 50.0 | 87.5 |
| Confirmed asbestos exposure (%) | 12.9 | 33.3 |
