# Supplementary material for: IL‐7 is expressed in malignant mesothelioma and has a prognostic value
Source: Mol Oncol. 2022 Sep 10;16(20):3606–19. doi: 10.1002/1878-0261.13310 (PMC9580880; doi:10.1002/1878-0261.13310)
Supplement: Supplementary file 17 — Table S5. Correlation of IL7R expression with markers of immune cells in TCGA database. [file MOL2-16-3606-s010.docx]

Table S5: Correlation of IL7R expression with markers of immune cells in TCGA database.

| Correlation *IL7R* | Spearman R | p |
| --- | --- | --- |
| CD68 | 0.303 | 4.271e-3 |
| CD163 | 0.476 | 3.172e-3 |
| CD206 | 0.453 | 1.069e-5 |
| IL10 | 0.393 | 1.663e-4 |
| CD4 | 0.451 | 1.182e-5 |
| FoxP3 | 0.485 | 1.983e-6 |
| CD8a | 0.581 | 3.69e-9 |
| CD274 | 0.462 | 6.572e-6 |
