# Supplementary material for: IL‐7 is expressed in malignant mesothelioma and has a prognostic value
Source: Mol Oncol. 2022 Sep 10;16(20):3606–19. doi: 10.1002/1878-0261.13310 (PMC9580880; doi:10.1002/1878-0261.13310)
Supplement: Supplementary file 18 — Table S6. Correlation of IL7 expression with markers of immune cells in TCGA database. [file MOL2-16-3606-s016.docx]

Table S6: Correlation of IL7 expression with markers of immune cells in TCGA database.

| Correlation *IL7* | Spearman R | p |
| --- | --- | --- |
| CD68 | 0.092 | 0.397 |
| CD163 | 0.216 | 0.486 |
| CD206 | -0.038 | 0.722 |
| IL10 | 0.110 | 0.348 |
| CD4 | 0.107 | 0.342 |
| FoxP3 | 0.182 | 0.919 |
| CD8a | 0.270 | 0.011 |
| CD274 | 0.272 | 0.010 |
